# Cystic mass of the right iliac fossa: don't forget about lymphatic malformation

**DOI:** 10.1259/bjrcr.20200165

**Published:** 2020-12-22

**Authors:** Habib Bellamlih, Ayman El Farouki, Rahal Mssrouri, Sabrine Derqaoui, Ahmed Jahid, Nabil Moatassim Billah, Ittimade Nassar

**Affiliations:** 1 Department of Radiology, University Hospital Center IBN SINA, Mohammed V-Souissi University, Rabat, Morocco; 2 Department of Surgery, University Hospital Center IBN SINA, Mohammed V-Souissi University, Rabat, Morocco; 3 Department of Pathology, University Hospital Center IBN SINA, Mohammed V-Souissi University, Rabat, Morocco

## Abstract

Lymphatic malformation or cystic lymphangioma is a benign tumour of the lymphatic vessels. It is more commonly reported among children and has polymorphic clinical presentations. The diagnosis is based on imaging but requires histological confirmation. The treatment of choice is surgical excision for the abdominal and symptomatic localization.

We report the case of a 30-year-old female who consulted for right iliac fossa pain mimicking an acute appendicitis. The physical examination revealed a slight tenderness in the right iliac fossa without fever or palpable mass. Though the biological screening was normal, the imaging exploration has revealed the presence of a multiloculated cyst located in the right iliac fossa at the ascending colon and measuring 15 cm. The mass matches with lymphatic malformation. Therefore, a laparoscopy was performed, and complete resection of the cystic tumor was accomplished with right hemicolectomy. The histologic exam has confirmed the diagnosis.

Colonic lymphatic malformation is a rare and benign tumour, requiring a complete surgical excision to minimise any recidivism. The definitive diagnosis remains histological.

## Clinical presentation

A 30-year-old female with no pathological history consulted for acute pain in the right iliac fossa without fever or transit disorders. Physical examination has revealed a slight tenderness in the right iliac fossa without palpable mass. The clinical presentation suggested the diagnosis of an acute appendicitis. The biological screening was normal.

## Investigations/Imaging findings

**Figure 1.**
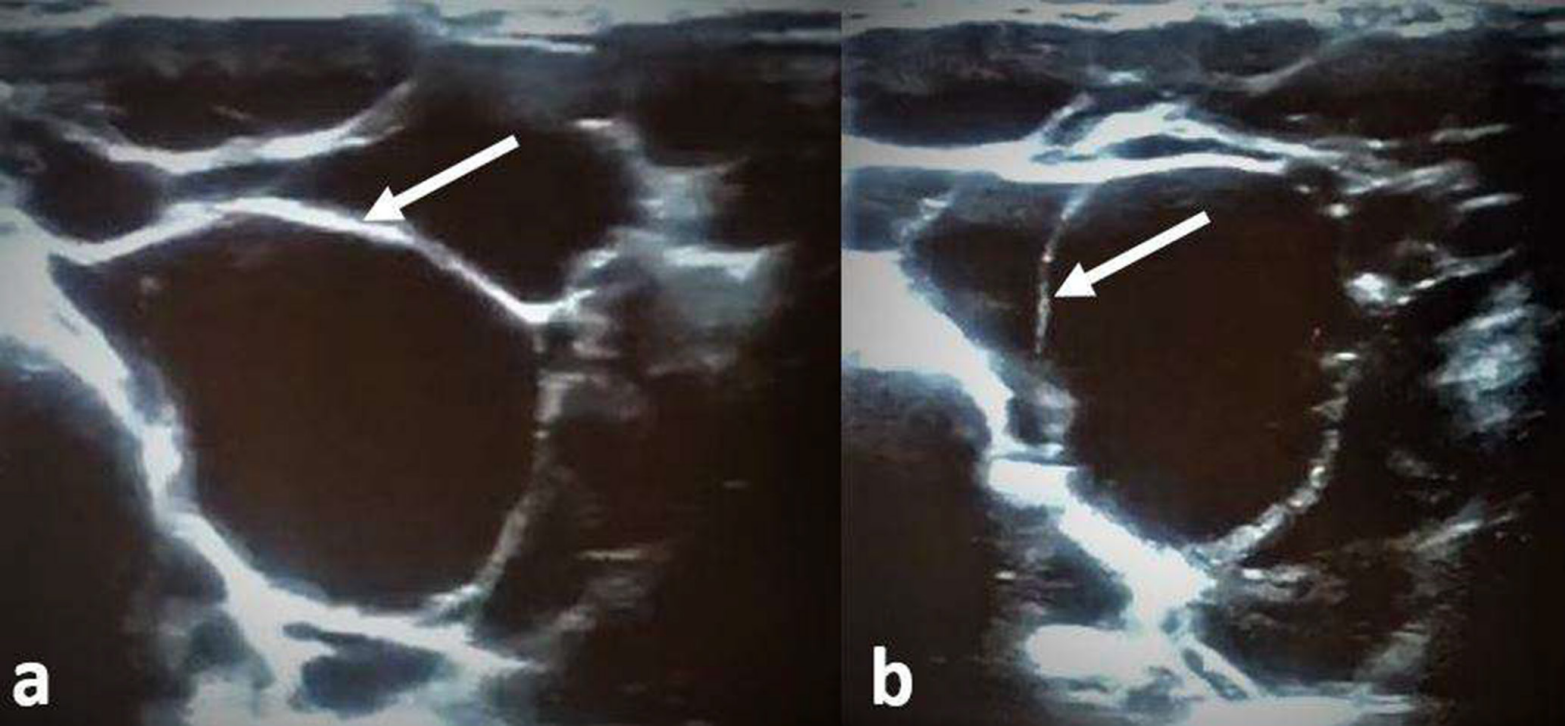
(a, b) Abdominal ultrasound showing a multiloculated cyst in the right iliac fossa containing a few thin septa (white arrows) without vascularisation in Doppler.

**Figure 2.**
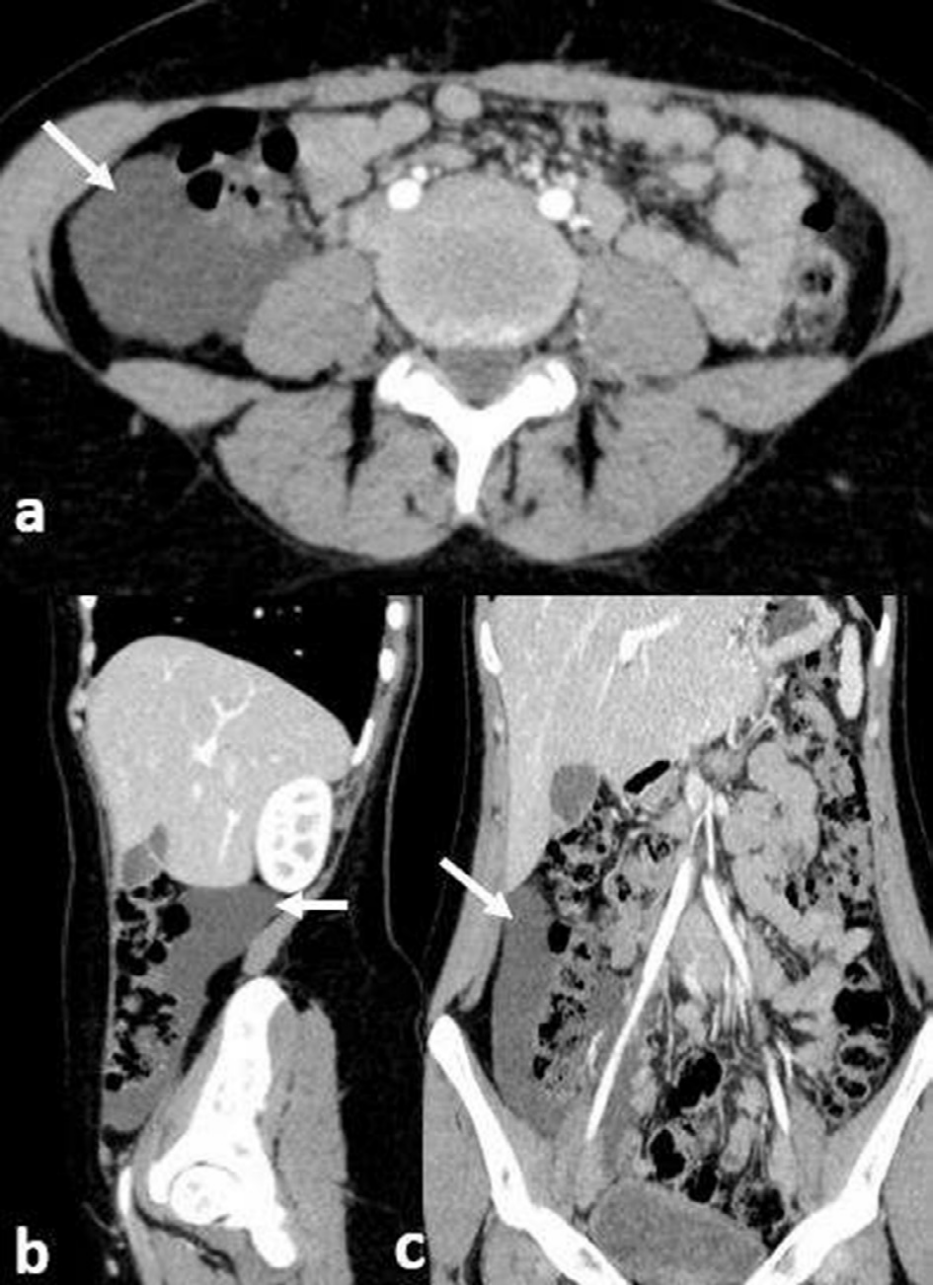
(a, b, c) Abdominal CT scan with i.v. contrast on axial plan, with sagittal and coronal reconstructions showing a multilocular cystic mass (white arrows) with a low-density and fluid density (4 HU), without enhancement neither visible wall, located at the ascending colon. HU, Hounsfield unit.

Abdominal ultrasound has showed a normal appendix (2 mm in mural thickness and 5 mm in maximal diameter) without infiltration of surrounding fat neither abdominal abscess. It also has objectified a multiloculated cyst in the right iliac fossa which measures 150 × 53 mm containing a few thin septa without vascularisation in Doppler (Figure 1). For better characterisation of the cystic mass, an abdominal CT scan with intravenous (i.v.) contrast was performed and documented a low-density, non-enhancing cystic mass with fluid density (4 HU), without visible wall, located at the ascending colon and measuring 15 cm. There were no signs of haemorrhage, rupture or volvulus (Figure 2).

## Differential diagnosis

The differential diagnosis includes a wide range of cystic intra-abdominal lesions, ranging from congenital duplication cyst, pancreatic pseudocyst, abdominal tuberculosis and abscess, hydatid disease, appendicular cyst, ovarian cyst, retroperitoneal cyst and malignancies such as mucinous carcinomatosis.^1,2^

## Treatment

**Figure 3.**
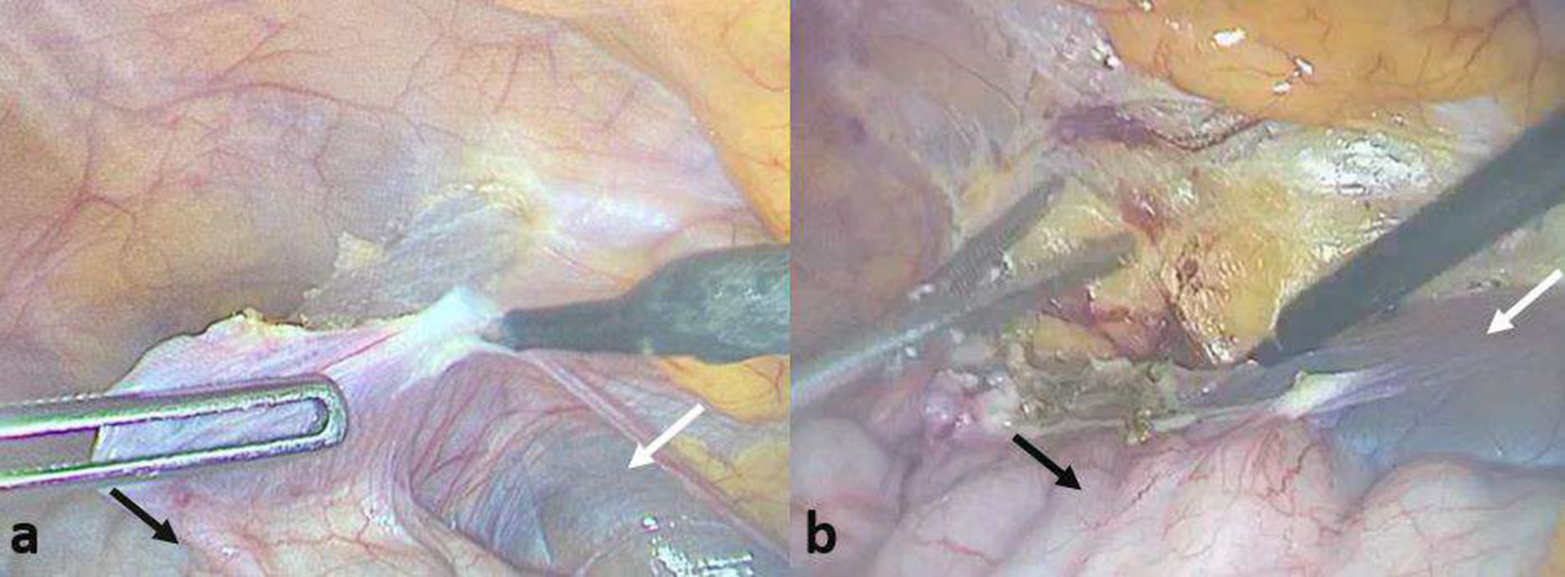
(a,b) Intra-operative images indicating a mutilocular cystic mass (white arrows) arising from ascending colon (black arrows).

**Figure 4.**
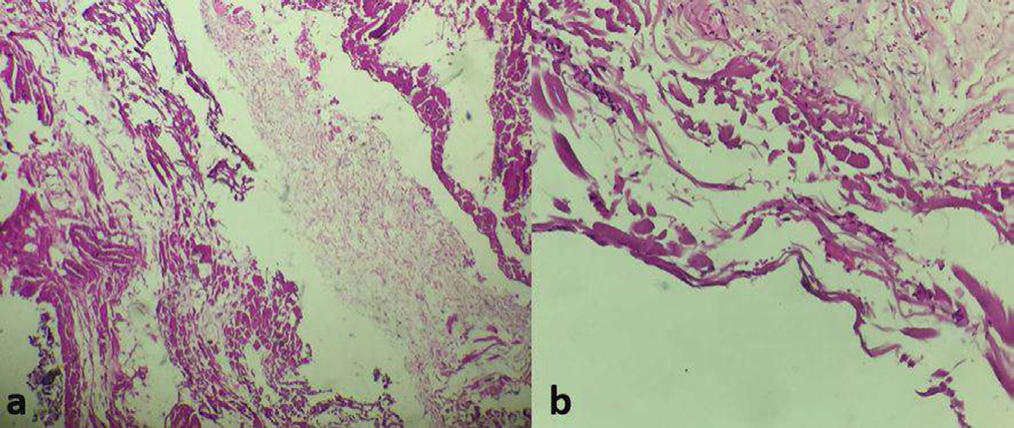
(a,b) Haematoxyline eosine stained sections showing cystic lymphangioma (a: low power x 200 b: High power x 400).

A laparoscopy was performed, and the exploration found a cystic mass that measured 15 cm of diameter (Figure 3). A complete resection was done with right hemicolectomy and the cystic mass was sent for a histological exam. On gross, the specimen consisted of a well-demarcated mass, measuring 150 × 50 mm. At sectioning, it was grey white multicystic appearing with serous type fluid. The histological analysis has revealed multiple cavities lined by endothelial bland cells and a fibrous stroma (Figure 4). Thus, the diagnosis of lymphatic malformation (LM) was retained.

## Discussion

LM are rare and benign tumours, typically arising in the axilla, head and neck regions in the paediatric population. These cystic hygromas develop rarely, in other regions.^3^

It does not often affects the intestinal tract and most of them settle in the mesentery, omentum, mesocolon and retroperitoneum.^4^

The incidence is about 0.18%. The LMs that appear in the wall of the intestine are considered even rarer and represented less than 1% of all types of lymphatic malformations.^5–7^

Colonic lymphatic malformation (CLM) was first reported by Chisholm and Hillkowitz in 1932, and is a submucosal tumour covered with normal mucosa. It occurs mainly among children.^8^

The specific aetiology of this tumour remains unclear. It may be due to a congenital defect of connection between the primary lymphatics spaces and the central collector system.^9,10^

The symptoms are generally poor, and non-specific. The CLM in paediatric patient are usually symptomatic if compared with adults, because children have smaller cavity which might induce frequent compressive symptoms. The most common symptom is the abdominal pain which mimics acute abdomen due to intra-abdominal bleeding, acute appendicitis, volvulus and bowel obstruction, ischaemia or perforation.^11^

The abdominal radiograph might show colonic distension, collapsed distal colon and even pneumatosis coli when complicated bowel obstruction or ischaemia was noted.^2^

Ultrasound is considered the first level of imaging investigation for a suspected mass suggestive of LM, thanks to its non-invasiveness, low cost, accessibility, and possibility of antenatal diagnosis: it grants not only the identification of the lesion and the definition of the cystic type structural characteristics, but its size also. The mass appears as a cystic liquid lesion, multilocular, thick wall without vascularisation in Doppler.

Usually, this technic needs to be integrated with CT scan or MRI in order to specify the morphology of the lesion, its components, the contrast enhancement, and the locoregional spread.^12^

For MRI, it seems to be interesting giving its capacity to make the differential diagnosis from other cyst-like masses but also its ability to characterise the various components of the lesion in particular the fatty content.^12^

Given its availability, the CT scan is considered the golden exam for establishing a pre-operative diagnosis. The LM appears as a well-limited mass with a homogeneous content, thin partitions without significant enhancement by the liquid contrast.

The content of the cystic masse can be heterogeneous either by the fatty component which appears as negative density or by the high density in case of haemorrhage.^13^

Definitive diagnosis remains mainly on histology. In fact, LM are classified into three types: capillary, cavernous and cystic, with the latter being the most common subtype. Macroscopically, LM present as yellow, greyish, or yellow-pink, multilocular cysts or spongy masses, containing watery/milky fluids at sectioning.^7^

On histology, LM consists of enlarged irregular spaces lined by flattened, epithelial cells devoided of atypia. The stroma is often fibrocellular. The presence of lymphocyte aggregates, smooth muscle bundles and adipocytes represent uncommon features. The diagnosis might require immunohistochemistry, endothelial cells stain positive with lymphatic endothelial markers such as D2 - 40 marker and also for vascular markers including CD34, CD31, and factor VIII–related antigen.^14–17^

Various forms of treatment have been described. A therapeutic abstention with a regular follow-up are recommended in case of accidental discovery of an asymptomatic CLM. A spontaneous regression can be obtained and it is observed in 1,6–16% of cases.^18,19^

A total surgical excision is actually a first choice treatment to avoid recidivism.^9^

A conservative approach should be performed for the adjacent organs because of the benign character of CLM.^19^

For symptomatic lesions that are accessible and unresectable, the aspiration of the cyst content or the percutaneous sclerosis with sclerosing products can be effective alternatives to surgical treatment with good results.^20–22^

## Learning points

- Colonic lymphatic malformation is a rare and benign tumor, requiring a complete surgical excision to minimise any recidivism.
- Colonic lymphatic malformation localised in the iliac fossa can be discovered in an emergency context and mimics acute appendicitis.
- The radiological imaging is essential for the pre-operative diagnosis of lymphatic malformation.
- The definitive diagnosis of lymphatic malformation is histological.
